# Antioxidant, Antidiabetic, and Anticholinesterase Activities and Phytochemical Profile of *Azorella glabra* Wedd

**DOI:** 10.3390/plants8080265

**Published:** 2019-08-03

**Authors:** Immacolata Faraone, Dilip K. Rai, Daniela Russo, Lucia Chiummiento, Eloy Fernandez, Alka Choudhary, Luigi Milella

**Affiliations:** 1 Department of Science, University of Basilicata, V.le dell’Ateneo Lucano 10, 85100 Potenza, Italy; 2 Spinoff BioActiPlant s.r.l., University of Basilicata, V.le dell’Ateneo Lucano 10, 85100 Potenza, Italy; 3 Department of Food BioSciences, Teagasc Food Research Centre Ashtown, Dublin D15KN3K, Ireland; 4 Department of Crop Sciences and Agroforestry, Faculty of Tropical AgriSciences, Czech University of Life Sciences, Praha 6 - Suchdol, Kamýcká 129, 165 00 Prague, Czech Republic

**Keywords:** Apiaceae, *Azorella glabra*, DPPH, *Beta*-Carotene Bleaching, RACI, phenolic characterization, UHPLC-MS/MS, polyphenols, flavonoids, health-promoting compounds

## Abstract

Oxidative stress is involved in different diseases, such as diabetes and neurodegenerative diseases. The genus *Azorella* includes about 70 species of flowering plant species; most of them are commonly used as food and in particular as a tea infusion in the Andean region of South America in folk medicine to treat various chronic diseases. *Azorella glabra* Wedd. aerial parts were firstly analyzed for their in vitro antioxidant activity using different complementary assays. In particular, radical scavenging activity was tested against biological neutral radical DPPH; ferric reducing power and lipid peroxidation inhibitory capacity (FRAP and *Beta*-Carotene Bleaching tests) were also determined. The Relative Antioxidant Capacity Index (RACI) was used to compare data obtained by different assays. Then, the inhibitory ability of samples was investigated against *α*-amylase and *α*-glucosidase enzymes involved in diabetes and against acetylcholinesterase and butyrylcholinesterase enzymes considered as strategy for the treatment of Parkinson’s or Alzheimer’s diseases. Moreover, the phytochemical profile of the sample showing the highest RACI (1.35) and interesting enzymatic activities (IC_50_ of 163.54 ± 9.72 and 215.29 ± 17.10 μg/mL in *α*-glucosidase and acetylcholinesterase inhibition, respectively) was subjected to characterization and quantification of its phenolic composition using LC-MS/MS analysis. In fact, the ethyl acetate fraction derived from ethanol extract by liquid/liquid extraction showed 29 compounds, most of them are cinnamic acid derivatives, flavonoid derivatives, and a terpene. To the best of our knowledge, this is the first report about the evaluation of significant biological activities and phytochemical profile of *A. glabra*, an important source of health-promoting phytochemicals.

## 1. Introduction

*Azorella glabra* Wedd., also known as *Azorella diapensioides* or *yareta*, is an endemic Bolivian species belonging to the Apiaceae (Umbelliferae) family. In the Andean region of South America, the plants belonging the genus *Azorella* are commonly used to treat several chronic diseases in folk medicine [1]. The *Azorella* genus is rich in diterpenoids, with mulinane and azorellane skeletal, compounds with a variety of important biological activities [2] that could explain the traditional use of the native food plant species and health benefits of the infusions. Moreover, in a previous study, *A. glabra* was suggested as the “future in the past” [3] for its dose and time dependent anti-proliferative effect on the multiple myeloma cells (MM), but is not present in any South American pharmacopoeias. In particular, the chloroform and *n*-hexane fractions were the most active and their cytotoxic effect could be attributed to the greater content of terpenoids. The influence of polarity of the solvents on total polyphenolic, flavonoid, and terpenoid contents was also reported. In fact, the highest total polyphenolic and flavonoid contents were reported in fractions obtained with polar solvents. On contrary, the highest total terpenoid content was reported in non-polar fractions. Further research studies are needed to explore the biological activities of *A. glabra* regarding its phytochemical composition to support its potential effects on the human health.

In this study, the antioxidant activity of *A. glabra* samples were ascertained by using three different complementary assays, namely 2,2-diphenyl-1-picrylhydrazyl radical (DPPH), Ferric Reducing Antioxidant Power (FRAP), and lipid peroxidation by *Beta*-Carotene Bleaching test (BCB) in addition to the previously radical scavenging activity against synthetic 2,20-azino-bis(3-ethylbenzothiazoline-6-sulfonic acid) (ABTS) and physiological superoxide anion (SO) and nitric oxide (NO) radicals [3]. The oxidative stress has been implicated in different diseases such as ageing, neurodegenerative disorders (Alzheimer’s disease and Parkinson’s disease), and diabetes [4,5]. For these reasons, we focused our attention also on the investigation of acetylcholinesterase (AChE) and butyrylcholinesterase (BChE) enzymes inhibition, the two enzymes involved in regulation of ACh levels in brain, therapeutic targets to cholinergic deficit [4]. Moreover, we evaluated the inhibition of *α*-amylase and *α*-glucosidase enzymes, an effective strategy to lower the levels of postprandial hyperglycemia typical in diabetic patients [6]. Then, the phytochemical profile was performed by LC-ESI-MS/MS analysis and the identification and quantification of polyphenols were achieved using commercially available standards [7]. To the best of our knowledge, this is the first report about the evaluation of biological activity, including antioxidant, antidiabetic, and anticholinesterase activities, and phytochemical profile of *A. glabra*.

## 2. Results and Discussion

The aerial parts of *A. glabra* were extracted by exhaustive dynamic maceration (four times for 3 h each) using 96% ethanol [7] with an extraction yield of 9.01%. To our knowledge, this is the first time that *A. glabra* was extracted by 96% ethanol, usually other species of the *Azorella* genus were extracted by petroleum ether [8,9]. Then, the present compounds in ethanol extract (Ag EtOH) were separated on the basis of the solvent affinity by liquid/liquid extraction using an increasing solvent polarity obtaining the extraction yields shown in Table 1, as reported previously [3].

### 2.1. Antioxidant Activity

The measurement of antioxidant activity on foods and plants are performed by more than one in vitro method in order to establish the antioxidant ability of samples [7]. For this reason, the antioxidant activity of the ethanol extract of *A. glabra* and its fractions were analyzed using three different complementary in vitro antioxidant assays.

The neutral DPPH radical was used to evaluate the radical scavenging activity. The samples were able to reduce the radical DPPH to the yellow coloured diphenylpicrylhydrazine in a concentration dependent manner. The AgEA showed the highest radical scavenging activity (Table 2) with 240.33 ± 10.73 mg TE/g value, followed by AgB. Instead, the lowest activity was found in AgC, and AgH was not active. Highly significant results were reported by Lamorte et al. for the radical scavenging activity of *A. glabra* samples against cationic (ABTS) and physiological (superoxide anion) radicals [3].

The FRAP test was used to evaluate the ferric reducing antioxidant power of samples and AgEA presented the highest FRAP value (410.29 ± 5.69 mg TE/g) followed by AgB (318.57 ± 2.77 mg TE/g). AgC and AgH were again the least active (Table 2).

The inhibition of lipid peroxidation was evaluated by the *β*-Carotene Bleaching assay (BCB) and the most active sample was again the AgEA (34.93 ± 1.37% AA), whilst the other fractions had similar BCB values. As expected, there was no BCB activity for the polar AgW fraction (Table 2).

**Figure 1:**
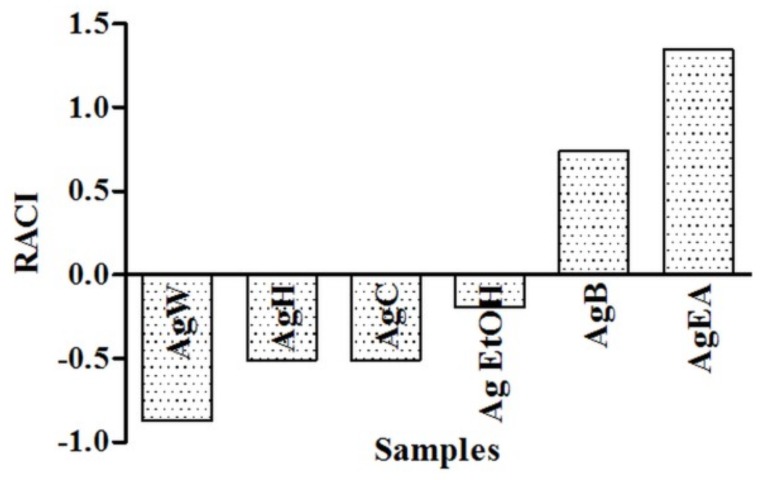
Relative Antioxidant Capacity Index (RACI) of *Azorella glabra* samples. Samples are crude ethanol extract (Ag EtOH), *n*-hexane fraction (AgH), chloroform fraction (AgC), ethyl acetate fraction (AgEA), *n*-butanol fraction (AgB), and water fraction (AgW).

The Relative Antioxidant Capacity Index (RACI) was calculated to integrate the results of the antioxidant activity obtained by DPPH, FRAP, and BCB assays in order to compare the different phytocomplex antioxidant ability [3,7]. The results obtained so far agreed with RACI values (Figure 1). In particular, the RACI evidences the ethyl acetate fraction presented the highest value (1.35), followed by the butanol fraction (0.74). The AgW fraction presented the lowest index (−0.87) and, therefore, a relative lack of antioxidant activity.

These antioxidant data are in line with other complementary antioxidant assay, i.e., ABTS [3]. Except Lamorte et al., there are no other studies that reported the antioxidant activity of *A. glabra*. Although other species of *Azorella,* in particular the antioxidant activity of *Azorella madreporica* aerial parts, was evaluated [10]. The aerial parts of *A. madreporica* were extracted with petroleum ether followed by methanol. Then, the methanolic extract was subjected to Total Polyphenolic Content (TPC), Total Flavonoid Content (TFC), and DPPH assays. *A. madreporica* had shown potent antioxidant property on DPPH radical (IC_50_ of 96.57 ± 1.00 μg/mL) and showed higher inhibition than Ag EtOH extract obtained in this study (IC_50_ of 1109.04 ± 33.84 μg/mL), which may be due to isoflavonoids identified by HR-ESI-ToF-MS [10]. These results exemplify how different extraction solvents (ether and methanol for *A. madreporica* and ethanol for *A. glabra*, respectively) influence the various in vitro protocols used to determine the radical-scavenging activity.

The RACI values illustrated in Figure 1 may explain the high total polyphenol content of the ethyl acetate and butanol fractions and the correlation between TPC and antioxidant activity in the earlier study [3]. Moreover, the fractions obtained by polar solvents (ethyl acetate and *n*-butanol) reported the highest total polyphenol content, indicating that the majority of polyphenolic compounds in the aerial parts of *A. glabra* could be of polar nature [11].

### 2.2. Determination of Anticholinesterase Activity of A. glabra Samples

**Figure 2:**
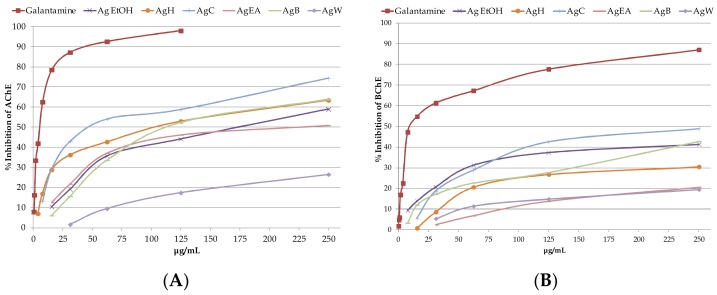
Acetylcholinesterase (AChE) (**A**) and butyrylcholinesterase (BChE) (**B**) inhibition activity of galantamine and *Azorella glabra* samples. Samples are galantamine, crude ethanol extract (Ag EtOH), *n*-hexane fraction (AgH), chloroform fraction (AgC), ethyl acetate fraction (AgEA), *n*-butanol fraction (AgB), and water fraction (AgW). Data are mean ± standard deviation from three experiments performed in triplicate.

The *A. glabra* samples had a concentration-dependent activity on acetylcholinesterase and butyrylcholinesterase enzymes (Figure 2). In particular, the AgC and AgH fractions showed a moderate activity by in vitro AChE assay (IC_50_ of 30.75 ± 0.67 and 99.19 ± 6.18 μg/mL), compared with the positive control galantamine (IC_50_ of 4.68 ± 0.31 μg/mL) (Table 3). BChE enzyme was inhibited only by Ag EtOH extract, and AgC and AgB fractions that showed butyrylcholinesterase activity weaker than galantamine (IC_50_ 16.07 ± 1.04 μg/mL). Again, the AgC fraction gave the lowest inhibition concentration (IC_50_ 240.28 ± 8.91 μg/mL) (Table 3).

This is the first report on anticholinesterase activity of *A. glabra*. In a previous study, the effect on the AChE enzyme of several terpenes (three lanostane-, two cycloartane-type triterpene, and two mulinane-type) isolated from *Azorella trifurcata* was reported. All compounds showed moderate inhibitory activity toward the enzyme, which may be due to the presence of acetate groups in these diterpenes [8].

### 2.3. Potential Antidiabetic Activity of A. glabra Samples

**Figure 3:**
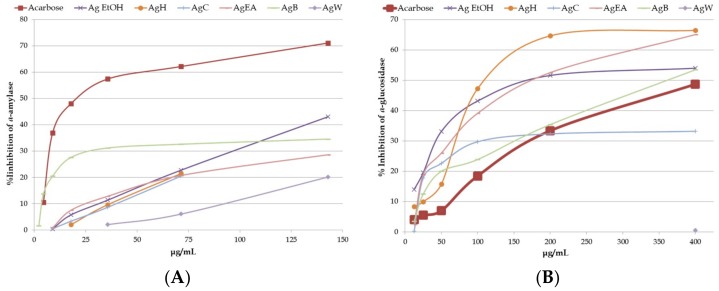
*α*-amylase (**A**) and *α*-glucosidase (**B**) inhibition activity of acarbose and *Azorella glabra* samples. Samples are acarbose, crude ethanol extract (Ag EtOH), *n*-hexane fraction (AgH), chloroform fraction (AgC), ethyl acetate fraction (AgEA), *n*-butanol fraction (AgB), and water fraction (AgW). Data are mean ± standard deviation from three experiments performed in triplicate.

The inhibition of *α*-amylase and *α*-glucosidase enzymes is an important strategy in the treatment of obese and/or diabetic patients. Different concentrations of the *A. glabra* samples were subjected for inhibitory activities of both the enzymes. The samples were concentration-dependent and acarbose was used as positive control (Figure 3).

In *α*-amylase inhibition assay, only the Ag EtOH reached the IC_50_ (172.25 ± 7.25 μg/mL), which was higher than acarbose (IC_50_ of 22.78 ± 0.29 μg/mL, Table 4).

Whereas in the *α*-glucosidase assay, all samples except for AgC and AgW fractions showed inhibition activity. Moreover, Ag EtOH, AgH, AgEA, and AgB fractions showed IC_50_ values lower than acarbose (IC_50_ of 401.15 ± 25.94 μg/mL) and the best of all were the AgH and AgEA fractions (IC_50_ of 159.91 ± 5.59 and 163.54 ± 9.72 μg/mL, respectively) (Table 4).

This is the first report on antidiabetic activity of *A. glabra*. A previous study on *Azorella compacta* had also shown a potent antidiabetic property [12]. The *Azorella* species has been used as antidiabetic medicine by the Andean natives. Fuentes et al. in 2005 [12] suggested that the diterpenic compounds mulinolic acid, azorellanol, and mulin-11,13-dien-20-oic acid isolated from *A. compacta* are responsible for the antidiabetic activity in ethnomedicine. It is also possible that the anti-diabetic properties of *A. compacta* is due to its ability to increase insulin secretion as mentioned in a review by Prabhakar and Doble [13].

### 2.4. Identification and Quantification of Phytochemicals

**Figure 4:**
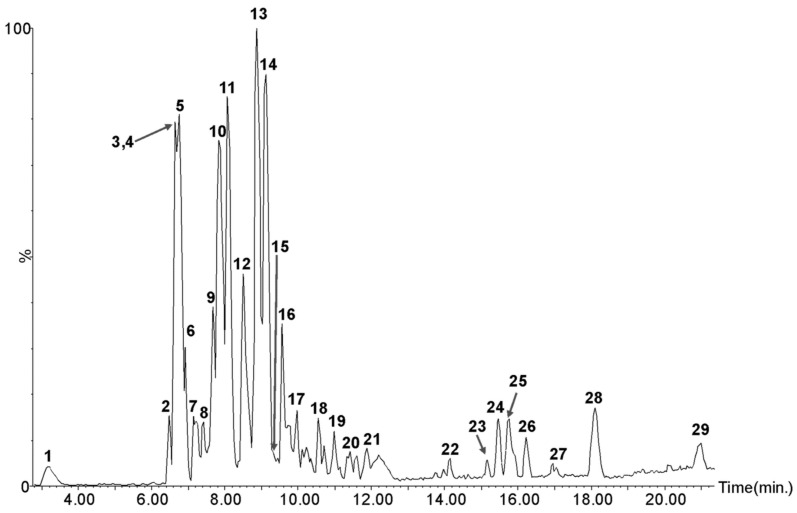
Ethyl acetate fraction of *Azorella glabra* base peak intensity (BPI) chromatogram.

In order to evaluate the compounds responsible for the various bioactivities examined above, the sample with the best antioxidant activity and the best enzyme inhibition was subjected to liquid chromatography-tandem mass spectrometry analysis (LC-MS/MS). The *A. glabra* ethyl acetate fraction (AgEA) was therefore chosen for the LC-MS/MS (Figure 4) as it showed the highest antioxidant activity, the best RACI value, and antidiabetic activity. In other species of *Azorella* genus were identified compounds belonging mainly to the class of diterpenoids [8,12], but this is the first report of phytochemical profile of *A. glabra*. The profile of AgEA obtained by LC–MS analysis is shown in Figure 4.

More than 29 compounds were detected and tentative identification of most of them was reached through accurate mass and fragmentation pattern and aided by the existing literature (Table 5). For the first time in *A. glabra*, 11 compounds were identified by comparing their retention times with those of the available commercial standards (Table S1). In particular, we performed calibration curves for both identified and used standards. The R^2^ values for all calibration curves were over 0.99.

These compounds are cinnamic acid derivatives (chlorogenic acid (**1**), chlorogenic acid methyl ester (**6**), cynarin isomer (**7**), 3,5-di-*O*-caffeoyl quinic acid (**10**) and 3,4-di-*O*-caffeoyl quinic acid (**11**)), flavones (iso-orientin (**4**), orientin (**5**), luteolin-7-*O*-glucoside (**8**) and luteolin (**15**)), flavonol (quercetin-3-*O*-glucoside (**3**)) and a triterpene (oleanolic acid (**29**)).

The flavone orientin and the cinnamic acid derivative 3,5-di-*O*-caffeoyl quinic acid were the most abundant (104.22 ± 4.01 mg/g DW and 44.70 ± 4.14 mg/g DW, respectively). The identified phenolic compounds are known for their antioxidant properties [14,15,16,17,18,19,20] that could explain the good antioxidant activity of ethyl acetate fraction of *A. glabra*. In particular, flavonoids such as luteolin and luteolin-glycosides have been reported to have anti-inflammatory and antioxidant activities. Moreover, plant species containing these compounds are known for their anti-inflammatory properties. The antioxidant abilities of flavonoids are widely acknowledged. The two antioxidant structural features of flavonoids are the presence of a B-ring catechol group and of a C2-C3 double bond in conjugation with an oxo group at C4. Luteolin (**15**) and its glycosides iso-orientin (luteolin 6-*C*-glucoside, **4**), orientin (luteolin 8-*C*-glucoside, **5**), and cynaroside (luteolin 7-*O*-glucoside, **8**) fulfil these two structural requirements and the antioxidant activity of these compounds has been related to their ability to scavenge reactive oxygen and nitrogen species. Moreover, luteolin could be developed as a cancer chemopreventive agent and be useful in cancer therapy to sensitize tumor cells to the cytotoxic effects of some chemotherapeutic drugs [21,22].

Four compounds were tentatively identified as feruloyl-caffeoyl quinic acid isomers (Table 5) with *m*/*z* 529.13 (**12**, **13**, **14** and **16**) and ion fragmentations at *m*/*z* 367, 349, 191, 179, 161, and 135 [23,24]. In addition, one compound was tentatively identified as chlorogenic acid glucoside (**9**) with *m*/*z* 515.14 and ion fragmentations at *m*/*z* 353, 191, 179, 173, 161, and 135.

## 3. Materials and Methods

### 3.1. Chemicals, Reagents, and Equipment

Solvents as ethanol, *n*-hexane, chloroform, ethyl acetate, *n*-butanol, hydrochloric acid, glacial acetic acid, methanol, and phosphoric acid were purchased from Carlo Erba (Milan, Italy). Acetonitrile and formic acid were purchased from Merck (Wicklow, Ireland).

2,2-Diphenyl-1-picrylhydrazyl (DPPH), 2,4,6-tripyridyl-*s*-triazine (TPTZ), iron (III) chloride (FeCl_3_*6H_2_O), *β*-carotene, linoleic acid, Tween 20, 5,5′-dithio-bis(2-nitrobenzoic acid) (DTNB), acetylcholinesterase (AChE) from *Electrophorus electricus* (electric eel, type VI-s, lyophilized powder, CAS number: 9000-81-1), acetylthiocholine iodide (ATCI), butyrylcholinesterase (BChE) from equine serum (lyophilized powder, CAS number: 9001-08-5), *s*-butyrylthiocholine chloride (BTCC), trizma hydrochloride (Tris-HCl), bovine serum albumin (BSA), potassium phosphate monobasic, sodium chloride, sodium hydroxide, sodium phosphate, potassium sodium tartrate tetrahydrate, *α*-amylase from hog pancreas (CAS number: 9000-90-2), 3,5-dinitrosalicylic acid, starch, 4-*p*-nitrophenyl-α-d-glucopyranoside, and *α*-glucosidase from *Saccharomyces cerevisiae* (CAS number: 9001-42-7), were purchased from Sigma-Aldrich (Milan, Italy). Standards as 6-hydroxy-2,5,7,8-tetramethylchroman-2-carboxylic acid (Trolox), butylhydroxytoluen (BHT), acarbose, galantamine, and Leucine-Enkephalin were purchased from Sigma-Aldrich (Milan, Italy) and Merck (Wicklow, Ireland), respectively. Standards for LC-MS/MS analysis (chlorogenic acid, chlorogenic acid methyl ester, cynarin isomer, 3,5-di-*O*-caffeoyl quinic acid, 3,4-di-*O*-caffeoyl quinic acid, iso-orientin, orientin, luteolin-7-*O*-glucoside, luteolin, quercetin-3-*O*-glucoside and oleanolic acid) were purchased from Extrasynthese (Genay, France). Water was deionized using a Milli-Q water purification system (Millipore, Bedford, MA, USA).

All spectrophotometric measurements were done in 96-well microplates or cuvettes on a UV–VIS spectrophotometer (SPECTROstar^Nano^ BMG Labtech, Ortenberg, Germany). LC-MS/MS analyses were performed on a Q-Tof Premier mass spectrometer (Waters Corporation, Milford, MA, USA) coupled to an Alliance 2695 HPLC system (Waters Corporation, Milford, MA, USA). Mass spectrometry quantification of the polyphenols was performed using Multiple Reaction Monitoring (MRM) experiments by Waters Acquity (Waters Corporation, Milford, MA, USA) ultra-high performance liquid chromatography coupled with tandem mass spectrometry (UHPLC-MS/MS).

### 3.2. Plant Material and Samples Preparation

A voucher specimen of aerial parts *A. glabra* was stored at the University of La Paz after the collection in Bolivia near the Aymaya population/community (18.45° S to 66.46° W; 3750 msnm), Bustillo province, Potosí department, Bolivia. Samples of the species are found in the herbal medicinal plants of the National University Siglo XX, Llallagua, Potosí, Bolivia. A total of 140 g of aerial parts of *A. glabra* were subjected first to dynamic maceration with 96% ethanol and then to liquid/liquid extraction, as previously reported by Lamorte et al. in 2018 [3], obtained the initial ethanol extract (Ag EtOH) and the *n*-hexane, chloroform, ethyl acetate, *n*-butanol, and water fractions (AgH, AgC, AgEA, AgB and AgW, respectively). These six samples were analyzed for their biological activities.

### 3.3. Antioxidant Activity

#### 3.3.1. Radical Scavenging Activity

The samples of *A. glabra* were tested for their radical scavenging capacity by in vitro DPPH assay.

The reaction between different sample concentrations (50 μL) able to reduce the radical DPPH (200 μL) to the yellow colored diphenylpicrylhydrazine was monitored by spectrophotometer at 515 nm after 30 min and quantified as milligrams of Trolox Equivalents per gram of dried sample (mg TE/g) [7,32,33].

#### 3.3.2. Ferric Reducing Antioxidant Power Assay (FRAP)

The reduction of the Fe^3+^ complex of tripyridyltriazine [Fe(III)(TPTZ)_2_]^3+^ to Fe^2+^ complex [Fe(II)(TPTZ)_2_]^2+^ (225 μL) by antioxidants present in acidic medium of different sample concentrations (25 μL) was monitored at 593 nm after 40 min at 37 °C and Trolox was used as reference antioxidant standard (FRAP values were expressed as mg TE/g) [7].

#### 3.3.3. *β*-Carotene Bleaching Assay (BCB)

This assay was used to evaluate the inhibition of lipid peroxidation. The *β*-carotene emulsion (950 μL) was mixed with sample at initial concentration of 1 mg/mL (50 μL) and BHT was used as positive control. The microplate with 250 μL of solution of each samples was placed at 50 °C for 3 h and the absorbance was measured at 470 nm at 0′, 30′, 60′, 90′, 120′, 150′, and 180′. The results were expressed as percentage of *β*-carotene bleaching inhibition (% Antioxidant Activity, % AA) [7].

### 3.4. Determination of Anticholinesterase Activity

The anticholinesterase inhibition of samples was investigated by Ellman’s reaction [4]. In the in vitro assays, acetylcholinesterase (AChE) and butyrylcholinesterase (BChE) catalyze the hydrolysis of acetyl- and butyryl-thiocholine, two alternative analogs of natural substrate acetylcholine. The quantification of free sulfhydryl groups was performed by 5,5′-dithio-bis-2-nitrobenzoic acid (DTNB) and the absorbance was measured at 405 nm for 2 min. The galantamine, a drug used to treat Parkinson’s and Alzheimer’s diseases, was used as positive control. The results were expressed on the basis of the concentration of the sample required to inhibit the activity of the enzyme by 50% (IC_50_) in μg/mL calculated by nonlinear regression analysis.

In particular, in AChE inhibition in vitro assay, different concentrations of sample (0.10–1000 μg/mL), buffer B (50.00 mM Tris-HCl, pH 8.00 containing 0.10% BSA), 3.00 mM DTNB, and 15.00 mM acetylthiocholine iodide were mixed and the reaction was started by adding 0.18 U/mL of AChE enzyme.

The BChE inhibition in vitro assay was performed in a similar way by using 15 mM butyrylthiocholine iodide as substrate and 0.10 U/mL of BChE enzyme [4].

### 3.5. Antidiabetic Activity

The enzymes involved in postprandial hyperglycemia are the *α*-amylase enzyme, which breaks down large and insoluble starch molecules into absorbable molecules and then in maltose, and the *α*-glucosidase enzyme that catalyzes the end step of digestion of starch and disaccharides. For this reason, the inhibitors of *α*-amylase and *α*-glucosidase enzymes were used in diabetic patients and the inhibitory ability of samples against both enzymes was screened.

#### 3.5.1. *α*-Amylase Inhibition

Different concentrations of each sample (0.50–300 μg/mL) were mixed to *α*-amylase enzyme from hog pancreas and incubated for 10 min at 25 °C. After pre-incubation, a substrate solution of 1% starch solution was added to the reaction mixture that was again incubated for 10 min at 25 °C. Then, the reaction was stopped by the yellow-orange 3,5-dinitrosalicylic acid color reagent and the vials were incubated for 10 min at 100 °C. Then, distilled water was added to the reaction mixture and the absorbance was measured at 540 nm as described by Milella et al. [6,34]. The acarbose, a clinical antidiabetic drug, was used as positive control and the results were expressed as IC_50_ in μg/mL on the basis of the concentration of the sample required to inhibit the activity of the enzyme by 50% calculated by nonlinear regression analysis.

#### 3.5.2. *α*-Glucosidase Inhibition

The ability of samples to inhibit the *α*-glucosidase enzyme was assessed using a previously reported procedure [6,34]. Different concentrations of sample (1–1600 μg/mL), phosphate buffer, 4-*p*-nitrophenyl-α-d-glucopyranoside, and *α*-glucosidase enzyme were mixed and incubated at 37 °C for 10 min. Then, the release of glucose and *p*-nitrophenol (yellow) by *α*-glucosidase enzyme was monitored spectrophotometrically at 405 nm. Again, acarbose was used as positive control and the results were expressed as IC_50_ in μg/mL.

### 3.6. Identification and Quantification by Liquid Chromatography Mass Spectrometry

The characterization of ethyl acetate of *A. glabra* was carried out on a Q-Tof Premier mass spectrometer (Waters Corporation, Milford, MA, USA) coupled to an Alliance 2695 HPLC system (Waters Corporation, Milford, MA, USA) and the quantification of compounds was used a Waters Acquity (Waters Corporation, Milford, MA, USA) ultra-high performance liquid chromatography coupled with tandem mass spectrometry (UHPLC-MS/MS) as described previously [7]. Electrospray mass spectra data were acquired on a negative ionization mode for a mass range *m*/*z* 100 to *m*/*z* 1000. In particular, an Atlantis T3 C18 column was used (Waters Corporation, Milford, USA, 100.00 × 2.10 mm; 3.00 μm particle size) at 40 °C. The mobile phases were 0.10% aqueous formic acid (solvent A) and 0.10% formic acid in acetonitrile (solvent B). The stepwise gradient from 10% to 90% solvent B was applied at flow rate of 300 μL/min for 25 min. Cone voltage and capillary voltage were set at 30 V and 3 kV, respectively. Argon was used as collision gas and the collision induced fragmentation (CID) of the analytes was achieved using 12 to 30 eV energy. The quantification of present compounds was performed on a Waters Acquity HSS T3 C18 column (2.10 × 100.00 mm; 1.80 μm particle size) using water containing 0.10% formic acid (mobile phase A) and acetonitrile containing 0.10% formic acid (mobile phase B). The following gradient program was carried out: 0–2.50 min 2% B, 2.50–3.00 min 10% B, 3.00–7.50 min 15% B, 7.50–8.50 min 35% B, 8.50–9.50 min 98% B, and 9.50–10.00 min 2% B at a flow rate of 0.50 mL/min. The injection volume for all the samples and the standards was 3.00 μL. All the standards in the concentration ranging from 0.01 to 50.00 μg/mL were dissolved in 80% methanol. The Multiple Reaction Monitoring (MRM) quantitative method was developed for each of the standard compound using the Waters Intellistart software, and the quantifications of the data were carried out using the Waters TargetLynx™ Software (Waters Corporation, Milford, MA, USA) [35]. The ionization source conditions were as follows: capillary voltage 3 kV, cone voltage 35 V, source temperature 150 °C, desolvation temperature 350 °C, desolvation gas flow 200 L/h, cone gas flow 50 L/h, and collision gas flow 0.10 mL/min.

### 3.7. Statistical Analysis

The data were expressed as mean ± standard deviation (SD) at least three independent experiments performed in triplicate. The correlation among used assays was verified by the calculation of *p* values by one-way analysis of variance (ANOVA) using GraphPad Prism 5 Software (San Diego, CA, USA). Only *p* values of 0.05 or less were considered significant. The R^2^ values for all calibration curves were over 0.99.

## 4. Conclusions

The biological activities demonstrated for *A. glabra* samples in this study might partially justify its ethnobotanical uses in Bolivian populations. In particular, the phytochemical profile of ethyl acetate fraction of *A. glabra* revealed the presence of compounds with antioxidant, anti-inflammatory, anti-tumoral activities [21,22]. In addition, it was previously reported that aerial parts of *A. glabra* reduced the cell viability, induced the apoptosis, and arrested the cell cycle on multiple myeloma cells in G0/G1 phase [3].

In conclusion, the combination of *A. glabra* extract, a “health food,” with common drugs may offer a significant advantage for therapeutic efficacy in several treatments as diabetes, neurodegenerative diseases, and cancer and it may have economic implications in the health and pharmaceutical fields.

## Figures and Tables

**Table 1:** Extraction yields of *Azorella glabra* ethanol extract and its fractions.

Samples	Extraction Yield (%)
Ag EtOH	9.01
AgH	31.52
AgC	44.50
AgEA	2.23
AgB	5.66
AgW	16.10

Samples are crude ethanol extract (Ag EtOH), *n*-hexane fraction (AgH), chloroform fraction (AgC), ethyl acetate fraction (AgEA), *n*-butanol fraction (AgB), and water fraction (AgW). Data are expressed as percentage (%).

**Table 2:** Results of DPPH scavenging activity, Ferric Reducing Antioxidant Power (FRAP), and *β*-Carotene Bleaching assay (BCB) of *A. glabra* samples.

Samples	DPPH (mgTE/g)	FRAP (mgTE/g)	BCB %AA
Ag EtOH	28.17 ± 2.32 ^a^	73.58 ± 0.71 ^a^	26.70 ± 0.61 ^a^
AgH	nc	18.79 ± 0.66 ^b^	22.50 ± 0.65 ^b^
AgC	5.94 ± 0.27 ^b^	15.45 ± 0.44 ^b^	22.18 ± 1.54 ^b^
AgEA	240.33 ± 10.73 ^c^	410.29 ± 5.69 ^c^	34.93 ± 1.37 ^c^
AgB	224.91 ± 4.84 ^d^	318.57 ± 2.77 ^d^	21.68 ± 0.57 ^b^
AgW	43.12 ± 1.23 ^e^	95.33 ± 3.86 ^e^	nc

Samples are crude ethanol extract (Ag EtOH), *n*-hexane fraction (AgH), chloroform fraction (AgC), ethyl acetate fraction (AgEA), *n*-butanol fraction (AgB), and water fraction (AgW). Data are expressed as means ± standard deviation from three experiments; mg TE/g = mg of Trolox Equivalents per gram of dried sample; % AA = percentage of Antioxidant Activity at initial sample concentration of 1 mg/mL; different superscripts in the same row indicate significant difference (*p* < 0.05); nc = not calculable.

**Table 3:** AChE and BChE inhibition by galantamine and *Azorella glabra* samples expressed as IC_50_ values in μg/mL.

Samples	AChE Inhibition (IC_50_)	BChE Inhibition (IC_50_)
Galantamine	4.68 ± 0.31 ^a^	16.07 ± 1.04 ^a^
Ag EtOH	193.81 ± 13.32 ^b^	421.50 ± 39.38 ^b^
AgH	99.19 ± 6.18 ^c^	nc
AgC	30.75 ± 0.67 ^d^	240.28 ± 8.91 ^c^
AgEA	215.29 ± 17.10 ^b^	nc
AgB	113.08 ± 5.18 ^c^	362.06 ± 28.60 ^b^
AgW	nc	nc

Samples are galantamine, crude ethanol extract (Ag EtOH), *n*-hexane fraction (AgH), chloroform fraction (AgC), ethyl acetate fraction (AgEA), *n*-butanol fraction (AgB), and water fraction (AgW). Enzymatic inhibition not calculable (nc). Data are mean ± standard deviation from three experiments performed in triplicate. The concentration of the sample required to inhibit the activity of the enzyme by 50% (IC_50_) in μg/mL was calculated by nonlinear regression analysis. In each test, the values with the same letter are not significantly different at the *p* < 0.05 level, 95% confidence limit, according to one-way analysis of variance (ANOVA).

**Table 4:** *α*-Amylase and *α*-glucosidase inhibition by acarbose and *Azorella glabra* samples expressed as IC_50_ values in μg/mL.

Samples	*α*-Amylase Inhibition (IC_50_)	*α*-Glucosidase Inhibition (IC_50_)
Acarbose	22.78 ± 0.29 ^a^	401.15 ± 25.94 ^a^
Ag EtOH	172.25 ± 7.25 ^b^	207.70 ± 2.56 ^b^
AgH	nc	159.91 ± 5.59 ^b^
AgC	nc	nc
AgEA	nc	163.54 ± 9.72 ^b^
AgB	nc	373.77 ± 29.84 ^a^
AgW	nc	nc

Samples are acarbose, crude ethanol extract (Ag EtOH), *n*-hexane fraction (AgH), chloroform fraction (AgC), ethyl acetate fraction (AgEA), *n*-butanol fraction (AgB), and water fraction (AgW). Data are mean ± standard deviation from three experiments performed in triplicate. Enzymatic inhibition not calculable (nc). The concentration of the sample required to inhibit the activity of the enzyme by 50% (IC_50_) in μg/mL was calculated by nonlinear regression analysis. In each test, the values with the same letter are not significantly different at the *p* < 0.05 level, 95% confidence limit, according to one-way analysis of variance (ANOVA).

**Table 5:** Liquid chromatography-tandem mass spectrometry (LC–MS/MS) of ethyl acetate fraction of *Azorella glabra*.

Peak No.	RT (min)	[M-H]^−^ Observed *m*/*z*	[M-H]^−^Calculated *m*/*z*	Molecular Formula	MS/MS	Tentative Identity	mg/g DW	References
1	3.17	353.0888	353.0873	C_16_H_18_O_9_	191, 173, 135, 127, 93, 85	Chlorogenic acid	7.12 ± 0.83	[25]
2	6.48	427.1980	427.1968	C_21_H_32_O_9_	367, 327, 297, 285, 179, 161, 135, 101, 73, 61, 59	Methyl chlorogenate derivative	nq	[26]
3	6.64	463.0859	463.0877	C_21_H_20_O_12_	300, 271, 255, 179, 151	Quercetin-3-*O*-glucoside	0.07 ± 0.00	[26,27]
4	6.68	447.0918	447.0927	C_21_H_20_O_11_	357, 339, 327, 311, 299, 297, 285, 269, 253, 191, 175, 149, 133, 109	Iso-orientin	13.22 ± 1.43	[28]
5	6.84	447.0910	447.0927	C_21_H_20_O_11_	357, 339, 327, 311, 299, 297, 285, 269, 253, 191, 175, 149, 133, 109	Orientin	104.22 ± 4.01	[28]
6	6.92	367.1038	367.1029	C_17_H_20_O_9_	191, 179, 161, 107	Chlorogenic acid methyl ester	12.33 ± 0.04	[26]
7	7.14	515.1180	515.1190	C_25_H_24_O_12_	353, 179	Cynarin isomer	0.15 ± 0.03	[29]
8	7.41	447.0921	447.0927	C_21_H_20_O_11_	285, 151	Luteolin-7-*O*-glucoside	0.65 ± 0.10	[30]
9	7.67	515.1411	515.1401	C_22_H_28_O_14_	353, 191, 179, 173, 161, 135	Chlorogenic acid glucoside	nq	
10	7.83	515.1197	515.1190	C_25_H_24_O_12_	353, 335, 191, 179, 161, 135	3,5-di-*O*-caffeoyl quinic acid	44.70 ± 4.14	[26]
11	8.07	515.1209	515.1190	C_25_H_24_O_12_	353, 179, 173, 135, 93	3,4-di-*O*-caffeoyl quinic acid	23.12 ± 1.64	[26]
12, 13, 14, 16	8.49, 8.86, 9.12, 9.56	529.1365	529.1346	C_26_H_26_O_12_	367, 349, 191, 179, 161, 135	Feruloyl-caffeoylquinic acid isomers	nq	[23]
15	9.32	285.0380	285.0399	C_15_H_10_O_6_	151, 133	Luteolin	0.39 ± 0.01	[25]
17	9.98	325.1651	325.1651	C_17_H_26_O_6_	281, 263, 235, 219, 203, 191, 151, 111, 83, 59	Unknown	nq	
18	10.55	853.4720	853.4738853.4679	C_48_H_70_O_13_C_55_H_66_O_8_	584, 513, 191, 179, 161, 135, 119, 113, 101, 89, 85, 71, 59	Caffeoylquinic acid derivative	nq	
19	10.97	649.3929	649.3952	C_36_H_58_O_10_	407, 191, 129, 113, 85, 75	Unknown	nq	
20	11.37	691.4073	691.4057	C_38_H_60_O_11_	631, 191, 113, 85, 95	Unknown	nq	
21	11.87	867.4739	867.4742	C_45_H_72_O_16_	513, 408, 333, 285, 191, 179, 173, 153, 139, 89	Unknown	nq	
22	14.14	391.1744	391.1757	C_21_H_28_O_7_	391, 347, 305, 287, 259, 245, 217, 165	Unknown	nq	
23	15.17	677.3729	677.3748	C_33_H_58_O_14_	415, 397, 279, 179, 161, 119, 101	Unknown	nq	
24	15.46	504.3098	504.3087	C_29_H_45_O_7_	279, 242, 224, 168, 153, 79, 59	Unknown	nq	
25	15.76	426.9764	426.9785	C_15_H_8_O_15_	407, 387, 293, 283, 255, 217, 81	Unknown	nq	
26	16.22	480.3083	480.3087	C_27_H_45_O_7_	255, 242, 224, 168, 153, 79	Unknown	nq	
27	16.95	579.3354	579.3381	C_28_H_52_O_12_	269, 255, 89	Unknown	nq	
28	18.10	553.3193	553.3165	C_33_H_46_O_7_	523, 345, 97, 84, 73	Unknown	nq	
29	21.16	455.3539	455.3525	C_30_H_48_O_3_	407, 377	Oleanolic acid	0.23 ± 0.05	[31]

Identification of compounds based on *m*/*z*, fragmentation pattern and retention time of standards. Quantities of the detected compounds were determined using commercial standards; nq = not quantified.

## Data Availability

All dataset used for this study is available on request.

## Supplementary Materials

The following are available online at https://www.mdpi.com/2223-7747/8/8/265/s1, Table S1: LC-Q-Tof chromatograms showing the retention times of standard compounds mix used for the identification and quantification of various polyphenols and a terpene in the ethyl acetate fraction of *Azorella glabra* Wedd.
