# Supplementary material for: Antioxidant, Antidiabetic, and Anticholinesterase Activities and Phytochemical Profile of Azorella glabra Wedd
Source: Plants (Basel). 2019 Aug 3;8(8):265. doi: 10.3390/plants8080265 (PMC6724412; doi:10.3390/plants8080265)
Supplement: Supplementary file 1 [file plants-08-00265-s001.pdf]

Table S1. LC-Q-Tof chromatograms showing the retention times of standard compounds mix used for the identification and quantification of various polyphenols and a terpene in the ethyl acetate fraction of *Azorella glabra* Wedd.

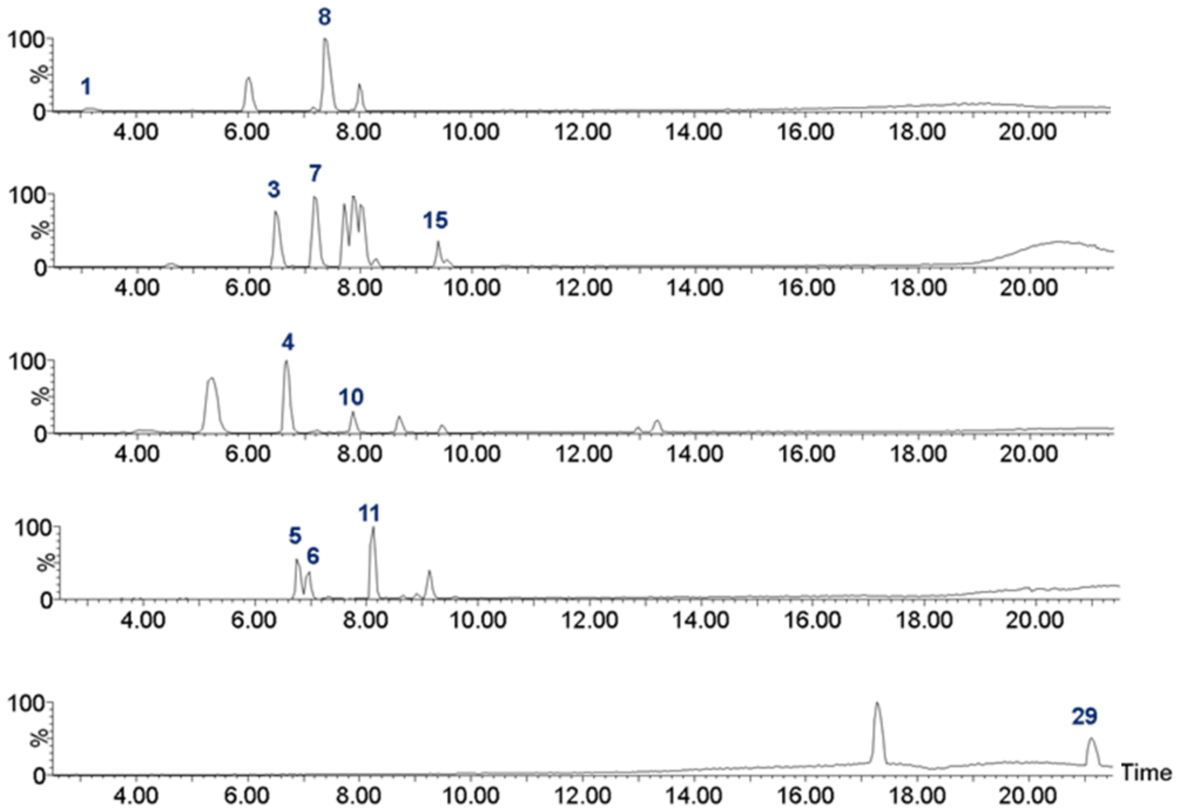

Identified compounds are chlorogenic acid (**1**), quercetin-3-*O*-glucoside (**3**), iso-orientin (**4**), orientin (**5**), chlorogenic acid methyl ester (**6**), cynarin (**7**), luteolin-7-*O*-glucoside (**8**), 3,5-di-*O*-caffeoyl quinic acid (**10**), 3,4-di-*O*-caffeoyl quinic acid (**11**), luteolin (**15**) and oleanolic acid (**29**)
